# Uncertainties around COVID-19 from the perspectives of oral health care workers during the first wave of SARS-CoV-2 infections in British Columbia, Canada

**DOI:** 10.1371/journal.pone.0249186

**Published:** 2021-04-22

**Authors:** Mario Brondani, Fernanda Almeida, Denise Cua, Tala Maragha, Kavita Mathu-Muju, Melody Shayanfar, HsingChi von Bergmann, Leeann Donnelly

**Affiliations:** 1 Department of Oral Health Sciences, Dental Public Health, Faculty of Dentistry, University of British Columbia, Vancouver, Canada; 2 Department of Oral Health Sciences, Faculty of Dentistry, University of British Columbia, Vancouver, Canada; 3 Department of Oral Biological and Medical Sciences, Faculty of Dentistry, University of British Columbia, Vancouver, Canada; 4 Department of Oral Health Sciences, Faculty of Dentistry, University of British Columbia, Vancouver, Canada; 5 Department of Psychology, Undergraduate Behavioural Neuroscience Student, Faculty of Science, University of British Columbia, Vancouver, Canada; Hofstra University, UNITED STATES

## Abstract

**Background:**

The first wave of COVID-19 infections caused disturbances in all aspects of personal and professional lives. The aim of this study was to explore the ways in which that first wave of novel coronavirus infections resulted in uncertainties, as experienced by members of the oral health care workforce in British Columbia, Canada.

**Methods:**

This qualitative inquiry purposefully recruited frontline oral health care workers, including dentists, dental hygienists, certified dental assistants, and administrative staff, via remote semi-structured interviews between April 20 and May 4, 2020. Coding, categories, and themes were inductively assigned.

**Results:**

A total of 45 interviews, lasting between 39 and 74 minutes each, were conducted involving 18 dentists (6 females), 12 dental hygienists (11 females), 6 certified dental assistants (all females), and 9 administrators/front-desk staff (7 females). Fifty-one hours of audio recordings and more than 650 single-spaced pages of transcripts were produced. Five main themes emerged pertaining to uncertainties surrounding COVID-19, patient care, personal lives and infectiousness, concern for the future, and variations among different pandemics. Certitudes were less evident, but surfaced mostly when considering a potential new normal resulting from the pandemic.

**Conclusion:**

Participants indicated that the uncertainties they felt were dependent upon what is known, and unknown, about the pandemic and the provision of oral health care during the first wave of infections. Future studies are needed to include the viewpoints of oral health care workers from other provinces, as well the perceptions of patients who received oral health care during the height of the first wave of the pandemic.

## Introduction

More than 110 million people around the world were infected with SARS-CoV-2 (severe acute respiratory syndrome coronavirus 2) by March 1, 2021, following the initial outbreak of the virus in Wuhan, China in December 2019 [1, 2]. Some of those infected individuals developed the novel Coronavirus Disease (COVID-19), and more than 2.5 million of them have already lost their lives to this disease following the first, second, and now third wave of infections caused in part by variants, despite vaccinations. Virus spread among populations has been associated with aerosolized transmission, droplets generated from speech, and sneezing or coughing, as well as contact with contaminated surfaces [3–5]. There are also potential sources for airborne transmission associated with aerosol-generating dental care procedures; therefore, by March 16, 2020, dental regulatory authorities across Canada strongly recommended the suspension of all routine and elective oral health care to contain the transmission of SARS-CoV-2 during the first wave of infections [6]. As the majority of Canadian dental offices were closed for preventive and clinical care, only emergency cases were addressed at that time–mostly related to pain relief and management of infection [7, 8]. The COVID-19 pandemic itself brought sweeping changes to the way oral health care is performed, leading to uncertainties among various members of the oral health workforce who are currently delivering oral health care [9]. Uncertainties appear to be “rooted in the incompleteness of knowledge” [10], and they are inherent to everyday clinical practice [11–14]. However, the extent to which the first wave of COVID-19 infections led to uncertainties in the professional and personal lives of dentists and dental hygienists is unknown. The objective of this study was to explore the ways in which the novel coronavirus outbreak resulted in uncertainties experienced by members of the oral health care workforce in British Columbia, Canada, during the first wave of infections in the Spring of 2020. The study presented herein was part of a larger project entitled “Structural preparedness during the COVID-19 pandemic and the provision of urgent oral health care”. As such, we are only presenting the findings pertaining to this study’s objective, which focuses on perceived uncertainties.

## Methods

Ethics approval was granted by the University of British Columbia’s Behavioural Research Ethics Board (# H20-01147). Written consent to participate was obtained from all participants. A qualitative inquiry engaged frontline oral health care workers, including dentists, dental hygienists, certified dental assistants, and staff, who either stopped the provision of oral health care or continued to work to treat dental emergencies after the curtailment of oral health care services in British Columbia on March 16, 2020. Participants were purposefully recruited from a province-wide professional email list or by word-of-mouth (snowball sampling) [15] and contacted the first author by email in April 2020. They were given the rationale for the study and its objectives, were informed about the interview process, and received an informed consent form via email. In addition, participants received a $150 honorarium in appreciation for their time. Interviews were scheduled at a date and time convenient for the participants between April 20 and May 4, 2020; face-to-face interactions did not occur, instead the interviews took place via phone, Zoom© video communication, or Skype Microsoft©. In-depth, semi-structured interviews were conducted in order to obtain even representation among the professions and roles, along with a fair distribution of gender (when possible), work location, and years of experience (Table 1). Upon mutual agreement, the first two interviews were conducted in the format of a conference call with numerous members of the research team (MB, TM, LD, KMM, HCvB) to calibrate and refine the main and probing questions. The interview guide included the following questions:

1) What do you know about the COVID-19 outbreak?

    *What do you know about the transmission of the virus?*

    *How can a patient be at risk for transmission?*

2) What do you understand about “preparedness” or “being prepared” to offer oral health care during the pandemic?

    *Can you give an example?*

    *How do you think we/you should prepare during the pandemic?*

3) Based on your own awareness and experiences, what actions and/or procedures would you like to see implemented (or that you are implementing) in order to resume care?

    *Can you give examples?*

    *How are you modifying your clinic infection control protocols to keep up with the new information that is constantly emerging about COVID-19?*

4) Is there anything else that still concerns you about the COVID-19 outbreak?

    *Why? Please explain*.

**Table 1:** Demographic information about the participants including profession, gender, years of experience, practice location, and working status.

Profession*	*Gender^*	*Years of Experience~*	*Location of Practice*^*<*^	*Office status*^*>*^	Working status **
**DMD1**	M	7	VCH	Office closed	Working
				Tele-dentistry	
**DMD2**	M	13	IH	Office opened	Working
				Emergency care	
**DMD3**	M	11	IH	Office opened	Working
				Emergency care	
**DMD4**	M	11	FH	Office opened	Working
				Tele-dentistry	
**DMD5**	F	12	IH	Tele-dentistry	On pandemic insurance
**DMD6**	M	11	FH	Tele-dentistry	Working
**DMD7**	F	3	IH	Office opened	Wage Subsidy
				Tele-dentistry	
**DMD8**	M	10	FH	Office closed	Working
**DMD9**	M	10	IH	Office closed	Working
				Tele-dentistry	
**DMD10**	F	30	NH	Office closed	On pandemic insurance
**DMD11**	F	11	NH	Office opened	Working
				Emergency care	
**DMD12**	M	32	FH	Office closed	On pandemic insurance
				Tele-dentistry	
**DMD13**	M	11	FH	Office opened	Working
				Emergency care	
**DMD14**	M	44	VIH	Office closed	On pandemic insurance
**DMD15**	F	1	VCH	Office opened	Working
				Emergency care	
**DMD16**	M	10	FH	Office opened	Working
				Tele-dentistry	
**DMD17**	F	3	VCH	Office opened	Working
				Emergency care	
**DMD18**	M	25	IH	Office closed	On pandemic insurance
				Tele-dentistry	
**DH1**	M	9	VCH	Office opened	CERB
				Tele-dentistry	
**DH2**	F	26	VCH	Office closed	On salary
**DH3**	F	32	NH	Office opened	On salary
**DH4**	F	25	VIH	Office closed	On salary
**DH5**	F	12	VCH	Office closed	EI
				Tele-dentistry	
**DH6**	F	22	IH	Office closed	On salary
				Tele-dentistry	
**DH7**	F	23	IH	Office closed	EI
**DH8**	F	29	IH	Office closed	CERB
**DH9**	F	23	VIH	Office opened	EI
				Tele-dentistry	
**DH10**	F	15	VIH	Office opened	CERB
				Tele-dentistry	
**DH11**	F	19	NH	Office opened	On salary
**DH12**	F	10	CVH	Office opened	On salary
**Admin1**	F	20	VCH	Office opened	On salary
				Tele-dentistry	
**Admin2**	M	27	VCH	Office closed	CERB
				Tele-dentistry	
**Admin3**	F	9	IH	Office closed	On salary
				Tele-dentistry	
**Admin4**	F	5	CVH	Office closed	Wage Subsidy
				Tele-dentistry	
**Admin5**	M	26	VCH	Office closed	On salary
**Admin6**	F	21	IH	Office closed	On salary
**FD 1**	F	22	VCH	Office closed	On salary
**FD 2**	F	12	VCH	Office opened	On salary
				Tele-dentistry	
**FD 3**	F	9	IH	Office opened	On salary
				Tele-dentistry	
**CDA1**	F	5	VCH	Office closed	EI
**CDA2**	F	11	VCH	Office opened	EI
				Tele-dentistry	
**CDA3**	F	9	VCH	Office opened	CERB
				Tele-dentistry	
**CDA4**	F	46	IH	Office closed	CERB
				Tele-dentistry	
**CDA5**	F	8	VCH	Office closed	CERB
**CDA6**	F	20	IH	Office closed	EI

*DMD: dentist; DH: dental hygienist; Admin: administration staff/clinic liaison; FD: front-desk staff; CDA: certified dental assistant.

^ M: male; F: female.

~ Working in that capacity.

^<^ Per each health authority region of British Columbia. VCH: Vancouver Coastal Health; FH: Fraser health; IHC: Interior Health; VIH: Vancouver Island Health; NH: Northern Health.

^>^ Office closed for patient care; Office opened for emergency care only; Tele-dentistry refers to cancelation and rebooking of patients, referrals, or over the phone prescription medication alone or in combination with emergency care or with offices that are closed.

** CERB: Canadian Emergency Response Benefit provides $500 a week for up to 16 weeks; EI: employment benefit provides a maximum amount of $573 per week for up to 45 weeks; Working status of dentists refers to either the provision of tele-dentistry only (likely without charging the patient) or of emergency care (likely billing the patient or the insurer); No hygienists were working even if their practices were open; Wage subsidy refers to a Government supported salary in which the employer pays 25% and the Government pays 75% of the salary resulting in a maximum payment per employee of $847 per week, for up to 3 months. Pandemic insurance pays up to $1,000 a day up to a $20,000 annual limit.

All interviews were audio recorded and transcribed verbatim for thematic analysis concurrent to data collection. While demographic data (e.g., gender, profession, work location, and years of practice) were recorded, a unique code was added to each de-identified transcript after information pertaining to gender was removed (Table 1). The de-identified transcripts were analyzed thematically between April 20 and May 30, 2020; an initial round of interactive analysis of the first five transcripts was performed to develop an audit trail and organize the coding scheme, then the remaining transcripts were analyzed independently by five of the authors (MB, TM, LD, KMM, HCvB). Coding refers to an inductive process of assigning a word or short phrase to a sentence or excerpt from the transcript. Each code refers to a specific idea or concept within the various segments of the transcript. Similar codes representing related ideas or concepts are grouped into categories or subthemes that are then clustered under a theme, as we have employed in previous studies [16–19]. Rigour was reached by employing reflexivity [20] during data collection and analysis, achieving data saturation [21], and conducting member checking [22] in order to reduce subjectivity and achieve the required standards for ethics and quality in qualitative research [23, 24]. Follow up interactions with the participants occurred via e-mail and/or a second round of interviews to clarify issues brought up in the first interviews; member checking was also offered to attest to the accuracy of our thematic analysis.

## Results

We recognized repetition of the responses after forty interviews, and it was decided to conduct at least one more interview with a representative from each participating group (e.g., dentist, dental hygienist, certified dental assistant, staff) to ensure that saturation had been achieved. The data collection process was then terminated resulting in 45 initial interviews lasting between 39 and 74 minutes each, and included information from 18 dentists (6 females), 12 dental hygienists (11 females), 6 certified dental assistants (all females), and 9 administrators/front desk staff (7 females). These 45 interviews led to 51 hours of audio recordings and more than 650 single-spaced pages of transcripts. Following the calibration coding of the first five transcripts, the previously mentioned five authors separately coded a different set of eight transcripts each. Although the authors independently coded each reflection, they met via conference calls to discuss the identified codes, categories, and themes, as well as to reach consensus on the thematic analysis process. In the context of the first wave of SARS-CoV-2 infections, five main themes emerged pertaining to uncertainties surrounding COVID-19, patient care, personal lives and infectiousness, concern for the future, and variations among different pandemics. These uncertainties surfaced during the first wave of a novel pandemic when information about the disease was still somewhat incipient and there was no vaccine in sight. Although the five themes might overlap in some respects, they are presented separately herein. For illustration purposes we used excerpts from the transcripts while including their respective participant demographic information for context.

### Uncertainties surrounding COVID-19

At the time of data collection social/physical distancing had already been highly encouraged in British Columbia for over 2 months. Due to the constantly evolving information available on the COVID-19 pandemic, uncertainties surfaced when participants were asked about the origins of the pandemic and which individuals were most at risk for contracting the disease. Participant DMD7, who at the time was providing dental emergency treatment only, told the interviewer that *“…as I saw on the news*, *it all started in China in 2019*, *around Christmas*, *from a live-animal meat market … and this novel Coronavirus seemed to spread through China and now to the world*. *It is transmitted via droplets with contaminated saliva*.*”* However, for Admin3, the issue of an airborne route for the spread of the virus was presented, but not fully understood.

In addition to relying on the news, others seemed to rely on less trustworthy sources of information leading to confusion and a sense of chaos. For example, CDA1 referenced a conspiracy theory circulating about the origin of the virus:

“*I heard a lot of things about the virus*, *I even read that it was created in a laboratory and that it got out of control*, *you know*, *and led to the turmoil we are living in*.*”*

Although the participant could not confirm the source of this understanding, she later recognized in the interview that *“we have to be careful with what is said on the news … not everything is the way it appears to be*, *or it is the sole truth for that matter*”.

In addition to the coronavirus’ origin and means of spread, uncertainty also exists around understanding the risk of getting infected with, or transmitting, SARS-CoV-2. In this context, the following exchange of information between the interviewer (I) and FD1 highlights the idea of COVID-19 being an equalizer:

“*FD1*: *I think it was around February or even March that I heard that this disease*, *or this infection*, *was like [an] equalizer*.

I: What do you mean by that?

FD1: It is like anybody can get it no matter if you are black or white, rich and poor. I understood [the disease] originated in China and now is spreading across the globe after people travelled. What I’m saying is that it can be an equalizer or equalize it in the society as I think it is affecting those that never travelled, as anybody can get it.”

However, to many other participants, including DH3, COVID-19 was not an equalizer because it *“is disproportionately impacting certain groups of people more than others*, *like those living in nursing homes*, *and the seniors who are dropping dead every day”*. And for DMD 15, *“I honestly fear that this disease will be much worse on [those] who were already suffering … will infect more the homeless*, *the very poor…sadly*.*”*

### Uncertainties surrounding patient care

As dental practices are considered high risk for SARS-CoV-2 transmission, practitioners had already ceased the provision of routine care for more than a month at the time of data collection. Those participants who were providing emergency care reported that they relied on different sources of information to make sense of the delivery of oral health care during this time. Some of the interviewees even relied on friends and colleagues to get the information they needed:

“*I’m getting a lot of information from my colleagues in Australia who are practicing*, *a lot of anecdotal evidence from what they’re seeing and also just from conversations with the other dentists I know that are practicing”* (DMD9).

Others were focused on the information they had yet to receive from the provincial associations as a trusted source for guidance:

“*We are waiting to receive that information from the [dental] association on how to proceed*, *what to buy in terms of Personal Protective Equipment [PPE]*, *what to do in terms of a proper protocol that has been sanctioned by them*. *Once we receive that information*, *we can take the necessary steps and avoid the sometimes-conflicting ideas and solutions that we are getting from other clinics–it gets messy”* (DMD14).

According to FD2, who was tasked with booking emergency patients for care: *“I feel like it is the blind leading the blind*, *really*. *I’m not sure if we*, *and them*, *know what to actually do properly*. *We are trying to follow the information*, *but it is too much*, *too often*, *and sometimes*, *too different*. *I get lost*.*”* This idea of somebody with limited knowledge getting advice from another person who may also be in the same position surfaced when DH10 was asked about PPE: *“I’m not sure*, *but I was told by a friend that she heard that N95 masks will be a new norm for us*, *and will be used at all times with patients*, *with the facial shield on top*.*”* Aside for uncertainties about how to safely provide urgent care, the participants were even more uncertain about their own lives when considering SARS-CoV-2 infection.

### Uncertainties toward personal lives and infectiousness

For many of the participants, their lives have been placed on hold and were not proceeding as planned. In the words of CDA4, who was receiving the Canadian Emergency Response Benefit (CERB):

“*My husband and I have a daughter in a facility and a mother in a seniors home*. *We have not been able to physically contact them to say hello with a hug or a touch to show how much we love them for over a month*. *That is disheartening for us*, *but at the same time*, *we do not want to infect them with this and make their lives even harder*.*”*

In fact, uncertainties involving the risk that the virus personally poses to participants made some unsure about their life plans; for DH3, her life was turned upside-down when she tested positive for SARS-CoV-2:

“*I went to see my doctor after not feeling well that day*, *and I knew what was coming*, *I just knew*. *So*, *I got tested and I came back home*. *The next day I went out as it was my son’s birthday*, *to get a few things at the grocery store*, *and then I got a call from them to say ‘you’re positive’*. *So*, *I had to cancel everything and then came the realization*: *who else have I infected during this time*? *It was not a good feeling*.*”*

And like DH3, other participants were also afraid of infecting their families and loved ones, given the risk they face as oral health care providers:

“*I need abundance of caution*. *I don’t want to get it [the virus]*, *I don’t want to get my family sick*, *I don’t want to get my sister’s family sick”* (DMD14).

“*How much protection do we need for ourselves*? *How much protection do we need for our patients*? *My husband does not want me to go back to work…if I get sick*, *I do not want my kids to get it*, *or my husband to get it”* (DH5).

### Uncertainties surrounding the future

While participants with a few decades of practice were uncertain about retirement, many younger participants, regardless of their experience, questioned their futures. The financial implications that incurred from curtailing services were also raised throughout the interviews. For DMD11 it was a matter of balancing new protective requirements for practice and quality of life:

“*I feel like there’s going to be fewer dentists because I don’t think people are gonna be able to do this*, *myself included*, *not only due to extra costs*, *but also because I don’t want to be all sweaty at work all day*, *I don’t want to have this indentation on my face from wearing a mask all day … if I can’t sit and chat with my patients*, *and have my mask off*. *If this is the new normal*, *I might want to do something else instead*.*”*

However, the decision to change professions may not be that easy or financially viable for some, as expressed by one participant whose son wants to apply for dental school:

“*…as an assistant you went to school for less than a year and perhaps you are not making a significant income*, *and maybe you might be looking at a career change*. *But to a dentist*, *that is different*. *They spent four years*, *actually more because you have to have another degree before becoming a dentist*. *And it’s quite expensive*. *So*, *you put all that effort*, *all that investment*, *and now you do not know how to pay for the debts*, *the education loans*, *and the costs of opening a clinic*. *That is not the same as for me*. *So*, *my son is now reconsidering”* (CDA3).

Furthermore, according to many other participants, there was the possibility of unanticipated benefits stemming from the pandemic. They foresaw oral health care reverting back to being more a matter of prevention and self-care at home, and less about aesthetics, if patients were to refrain from visiting a dental office as frequently as before the pandemic:

“*Maybe people now will rely more on their own self-care at home … it is the silver lining of this … people now paying closer attention [to] what they are doing [in terms of oral hygiene]”* (DMD14).

“*That’s what we should be doing at home … taking care of your teeth*. *Diet*, *exercise*, *and eating healthy kind of thing*. *And now if they don’t have as much access [to an oral health care provider]*, *they have to go back and actually pay attention to what they eat and how they brush”* (DH9).

“*In the future you’re going to see fewer people going into dental offices and you’re going to see people avoiding small restorations like a chipped tooth here and there*. *And they will figure ‘ok*, *I’ll just live with this’*, *you know*, *and that will not be a concern anymore*” (DMD10).

Furthermore, the first wave of infections has already taken a financial toll, as seen in Table 1. Many participants, mainly those who are typically employees, including dental hygienists, certified dental assistants, and front-desk staff, made use of the CERB (Canadian Emergency Response Benefit) or EI (Employment Insurance) funding that was available at that time, while others were laid-off. And for some dentists, who are typically business owners, the option was to make use of some form of pandemic insurance to cover some of their expenses, including overheads.

### Uncertainties surrounding variations among different pandemics

For those participants with over 30 years of experience, a natural comparison was drawn between SARS-CoV-2 and HIV, with participants circling back to their experiences from decades ago. While everyone recognized that these two infections were very different from each other, especially related to the route of transmission, it was reassuring that dentistry had already learned to adapt to yet another infectious agent. For Admin5, the parallel was around PPE:

“*When I first started*, *we didn’t use masks and gloves*, *and with a number of people you’re working on you had to hold your breath and run out to breathe*. *But that all changed—we were all tested for HIV*. *And we were very mindful on that and that’s when we started wearing glasses*, *and masks*, *and gloves*. *Now you would never consider working on somebody without any of it*.*”*

The concept of dentistry being done with bare hands during the pre-HIV era resonated with many other participants, including DH3, who commented that *“there were no gloves and there were no masks*. *But obviously HIV came … [and that is] when it became mandatory to wear gloves and a mask*.*”*

Another aspect of uncertainty involved divergence between AIDS/HIV and COVID-19 when considering the impact of both pandemics, as discussed by DMD14: *“back then*, *nothing shut down because of AIDS*. *The whole world didn’t shut down*, *right*. *I mean*, *it was definitely awful*, *but it wasn’t like the whole world pandemic that we now have with COVID-19*.*”* And according to DMD4, who also started practicing during the earlier HIV pandemic, transmission of the virus is vastly different:

“*What comes to mind is that HIV is a blood- and other body fluids-borne disease*. *It is almost impossible to get it in the dental office compared to this one [COVID-19] that is through your breathing*, *through saliva droplets which [are] everywhere*. *That’s the difference*.*”*

While parallels and differences can be drawn between HIV and SARS-CoV-2, the previous management of an infectious outbreak demonstrates that this new challenge can be surmounted to ensure that society receives oral health care. One of the participants, CD4, stated that *“we can learn from the past*, *as we did when HIV came*, *as we did when SARS came…we adapt*, *we change*, *we move past that*.*”*

Although this study focused on the uncertainties experienced by the participants in many aspects of their lives, a few offered some certitudes brought about by the pandemic and were more optimistic. Such optimism was shared mostly by those who took the initiative to develop their own protocols for clinical practices, which reaffirmed their assertions around patient care and the practice of dentistry:

“*There are a few offices that*, *as far as I can tell*, *don’t have any of the protocols*. *So*, *I basically said*, *"You’ve got to do this*. *And the onus is on the operator to do all this*.*" We’ve done the ABC’s*, *and we are ready*. *We are ready and we are providing emergency care while protecting the public”* (DMD2).

For others, reassurance came from the realization that the COVID-19 pandemic will eventually subside “*as it happened with AIDS and SARS*, *that are now manageable”* (DH2). And in the words of DMD11, *“I’m hopeful*, *really*, *and I stay positive because life is short and although the concern is real*, *I’m optimistic that life will get back on its course”*. A sense of certitude also surfaced when considering the new normal: *“I think we will be faced with a new norm*, *so our lives get some level of regularity once the panic and stress are over”* (Admin2).

## Discussion

This study explored the various ways in which the first wave of infections caused by the novel coronavirus outbreak led to feelings of uncertainty experienced by 45 oral health care providers and administrators in British Columbia, Canada, during the Spring of 2020. As the interviews took place during the first wave of infections, the findings have to be understood under that context–even if a second and third wave were assumed to happen, a vaccine was to be developed, and a protocol for care during a pandemic was presented [25] participants were not asked about these now pressing developments [26]. The uncertainties shared by the participants were dependent upon what was (un)known about the disease, what could be done to safely provide oral health care, to what extent the pandemic would impact participants’ lives, what the future of oral health care would look like, and how similar/dissimilar the outbreaks of HIV and SARS-CoV-2 were by the Spring of 2020.

Uncertainties, like those reflected in the participants’ responses, are not entirely surprising given that they “*permeate every aspect of clinical practice–from diagnosing and managing dental caries to predicting success for [a treatment]*” [10] and occur as early as during undergraduate dental education [27, 28]. The COVID-19 pandemic has produced other uncertainties, considering information has changed almost daily due to the rapid rate knowledge is obtained about this novel coronavirus. Such rapid availability of new information from multiple sources has led to many different interpretations, and even the misinterpretation of information, as was seen with the media debate around wearing facial coverings–including masks–in public. The idea of wearing facial coverings does not readily fit some existing preconceived notions in western cultures, but acceptance of the necessity of wearing masks during the pandemic seems to have become more mainstream in various contexts [29] while also becoming *“a catalyst for political conflict … viewed through a partisan lens”* [30]. Some participants appeared to gather information from primary sources, such as the Provincial Dental Associations, Centers for Disease Control (CDC), and World Health Organization (WHO) [6, 31], and seemed to be more confident in their knowledge and could clearly articulate their thinking. Participants who referenced other sources of information, including media and social circles, were less certain about the evidence they provided, perhaps due to confusion or cognitive dissonance over what they learned and what they believed, which ultimately reflected an incompleteness in their knowledge [10].

A number of interviewees mentioned the zoonotic source of SARS-CoV-2 likely originated from bats, more specifically the Chinese horseshoe bat (*Rhinolophus sinicus*), as reported by Chan and coworkers [32] and Lu and colleagues [19]. Other interviewees were uncertain about its origin, which has recently come into question since traces of COVID-19 were found in sewage samples from Spain, Italy, and Brazil that pre-date its discovery in China [33], and after the WHO exploratory investigation in Wuhan at the beginning of 2021 [34]. Participants also seemed to disagree about whom is at risk for COVID-19. While many believed that anyone can be at risk by referring to the equalizing effect of the pandemic, others questioned that assumption by mentioning that COVID-19 disproportionately impacts certain racialized groups, including African Americans in the United States [20] and Canada [21], and those of lower socio-economic status [35], which further contributes to health inequalities rather than equalization. Many participants mentioned older adults as another group at higher risk for COVID-19, when in fact it is the multiple comorbidities present in this group that leads to the higher risk of more severe complications from the disease when compared to their healthier or younger counterparts [27, 31, 36, 37].

The reorganization of clinical work and oral health services is not straightforward and led participants to experience a great deal of uncertainty. Their concerns around PPE are legitimized by the fact that oral health care professionals are at a higher risk for contracting SARS-CoV-2 than other professionals [38]. However, participants also realized that there are still many uncertainties and unknowns related to COVID-19, from the efficacy of the current infection control protocols [25, 39] to striking a balance between self-care and patient care in their daily practice [7]. Although it is unthinkable that in this day and age an oral health professional would provide care without wearing disposable gloves, eye protection, and masks at the bare minimum, participants did mention the bare-handed dental care provided before the human immunodeficiency virus (HIV) pandemic. These disposable items are part of PPE, and fall under the principles of universal–or standard–precautions or procedures (UPs) [40, 41]. UPs refer to an approach for infection prevention and control that treats every patient and their bodily fluids, including blood and saliva, as infectious for any blood-borne pathogens in order to minimize the risk of cross-infection among both patients and health care workers [42]. As voiced by the participants, PPE and UPs were introduced to mainstream use following the Acquired Immune Deficiency Syndrome (AIDS) outbreak in the 1980’s [43, 44]. Under normal circumstances the transmission of HIV within the walls of a dental practice is very improbable [45], while the risk of SARS-CoV-2 transmission via saliva droplets is considered extremely high since droplets are part of any dental procedure; this prompted various participants to be uncertain about providing safe care while minimizing the risk of transmission to themselves and their families. In fact, when questioned about the transmissibility of SARS-CoV-2 via aerosols, no uncertainty seemed to exist as all participants mentioned the respiratory droplets route [4, 5, 46, 47]. Interestingly, none of the participants commented directly on the contact transmission route (or community transmission), which also occurs via aerosolized saliva droplets. This route has been the main reason for physical distancing measures adopted by many countries in response to the pandemic due to the likely airborne transmission of the virus [48]; hence, the suggestion of face coverage when physical distancing is not possible [30]. Moreover, Meng and colleagues [39] highlighted that *“the standard protective measures in daily clinical care are not effective enough to prevent the spread of COVID-19”*, which further justified the many uncertainties participants had in terms of protection during the provision of oral health care, and their concerns around bringing the virus home. Furthermore, competing personal demands, including issues surrounding the care of children and grandparents voiced by many participants, might have likely exacerbated these uncertainties on top of eclipsing pressures related to maintaining a viable practice; such pressures were found to have personal, financial, and professional implications [7].

Participants also shared their uncertainties regarding the future of the profession, their lives, and the viability of a dental practice. Financial uncertainties surfaced as dental offices were either closed or only attending to urgent care [7, 9]. Lay-offs were experienced by some of those with employee status who were on a form of government financial benefit aid, either CERB or EI; while some dentists, as business owners, were using pandemic insurance. This uncertain economic and health care environment may further affect dental care delivery [49].

Moreover, changes brought about by the pandemic are inevitable. Nearly 30 years ago, Hardie concluded that “*it goes without saying that the members of any professional group are more likely to modify their behavior if they are provided with logical*, *rational reasons to enact the suggested change”* [50]. Such changes are likely being introduced now under new oral health care protocols, but it remains unclear which preparations during an outbreak are deemed necessary [51] or will remain after vaccination. Whether or not these changes will actually constitute a new normal, there is still an opportunity for the profession to innovate given the uncertainties encountered during this pandemic. In fact, Brondani and Donnelly (along with others [52]) have shared the ideas presented by undergraduate dental students who suggested a shift in the provision of oral health care to focus on less invasive and non-aerosol generating procedures, such as the use of silver diamine fluoride and tele-dentistry when applicable [27]. In addition, when considering the new normal, Profitt goes beyond the changes in treatment to also highlight changes in products and technology for the practice of oral health care, and in the healthcare system as a whole [53]. Although “*unanticipated benefits*” such as enhanced self-care at home were mentioned as a silver lining of the pandemic, there were potential financial and health implications when patients had to place their ongoing care on hold [49, 54].

While there might exist a culture of certainty in dentistry, particularly in undergraduate education [11], uncertainties do indeed abound in everyday clinical dental practice, from diagnosis to treatment planning and prognosis [10, 12–14, 27]. These uncertainties have also permeated our participants’ personal and professional experiences at a time when a novel coronavirus has caused a global pandemic and resulted in millions of infections and deaths, along with an unprecedented financial crisis. While some participants have alluded to a new normal during these uncertain times, the pandemic might also optimize self-reflection so we can collectively reshape the culture of the dental profession and foster stronger resilience.

This study aimed to achieve rigour through reflexivity, data saturation, and member checking [22, 23]. Reflexivity referred to how the research team described the contextual intersecting relationships between the participants and themselves, as addressed ahead when discussing the limitations of the study [55]. Saturation referred to the point during data collection where no new information was provided on the issue under investigation, and the data did indeed become repetitive within the 45 interviews [56]. Member checking occurred at different stages of the study, where participants were given the opportunity to read their transcripts, and/or the thematic analysis, and/or the final report [57]; however, no participants offered comments when prompted.

Despite the interesting findings, this study is not without limitations. Given the current preference of social/physical distancing to curb the spread of SARS-CoV-2, all interviews took place at-a-distance and likely caused the loss of a more personal connection [58]. Although we offered participants the opportunity to be involved in member checking, only four participants chose to receive the transcripts and none of them provided any actual feedback; this is comparable with our experiences in previous studies [15, 59]. The sole focus on British Columbia and the relatively small sample size prevents full generalization to all oral health care providers, even though the data was indeed saturated. The purposeful recruitment method, although targeted province-wide, was unlikely to have reached all professionals and staff in British Columbia. The participant honorarium may have been high for this type of study and might have attracted those who would express their ideas in a more socially desirable manner, that is, they responded based on what they thought the interviewer would like to hear, likely influencing the results. The mixing of different professional groups (dentists, hygienists, assistants, and so on) may have hindered the peculiarities of each group. Despite the potential personal and professional stressors to be discussed at the time, the interviewers allowed the participants to be at ease, openly share their thoughts, and probed for information only as needed. Given the inter-provincial differences in professional regulations, it is necessary to include the opinions of a larger, Canada-wide group of oral health care providers (not just those in British Columbia) in future studies. In addition, the perspectives of the patients themselves, as the recipients of oral health care during this pandemic, should also be heard. Lastly, the third wave of SARS-CoV-2 infections caused in part by the many variants in Canada and in many other countries as of Spring 2021, along with the introduction of mass vaccination [26, 60], would likely influence the results of this study if repeated.

## Conclusions

This study employed semi-structured interviews with a sample of 45 oral health care providers and administrators across British Columbia to explore the ways in which the current novel coronavirus outbreak led to uncertainties in the delivery of oral health care in the Spring of 2020. Participants indicated that these uncertainties were dependent upon what was known, and also unknown, about the pandemic, given the constantly evolving information related to COVID-19. Many participants felt they did not have clear direction on how to provide safe oral health care in the present circumstances, and described their lives as being on pause for the moment; they also sensed that a new normal for oral health care is in the making. Future studies are required to include a larger sample of oral health care providers across Canada who might have experienced the pandemic differently, as well as the perspectives from patients receiving oral health care during this time.

## Abbreviations

AIDS: Acquired Immune Deficiency Syndrome
BC: British Columbia
CDC: Centers for Disease Control and Prevention
CERB: Canadian Emergency Response Benefit
COVID-19: Coronavirus Disease 2019
EI: Employment Insurance
HIV: Human Deficiency Virus
PPE: Personal Protective Equipment
SARS-CoV-2: Severe Acute Respiratory Syndrome-Coronavirus-2
UPs: Universal Precautions
WHO: World Health Organization
